# Evolving role of semaglutide in NAFLD: in combination, weekly and oral administration

**DOI:** 10.3389/fphar.2024.1343587

**Published:** 2024-02-23

**Authors:** Evgenia Koureta, Evangelos Cholongitas

**Affiliations:** Laiko General Hospital, Medical School, National and Kapodistrian University of Athens, Athens, Greece

**Keywords:** NAFLD, NASH, semaglutide combination, weekly semaglutide, semaglutide orally

## Abstract

Non alcoholic fatty disease (NAFLD) is the most common chronic liver disease that is managed in the liver departments. It seems that the prevalence of the disease is rising worldwide and as it has the same pathogenetic pathways with metabolic syndrome, treatments that target components of the metabolic syndrome seem promising for the therapy of NAFLD as well. In this review we discuss the evolving role of semaglutide, which is a glucagon-like peptide-1 receptor agonist (GLP-1 RA) that has been already approved for the treatment of type II diabetes mellitus (T2DM) and obesity.

## Introduction

Although many trials worldwide have been conducted to find an effective and safe treatment, the recommendation for patients with nonalcoholic fatty liver disease (NAFLD), which is an entity with rising prevalence (Vernon et al., 2011; Younossi et al., 2016; Mundi et al., 2020), still constitutes of exercise and dietary modifications (Vilar-Gomez et al., 2015; Kenneally et al., 2017; Romero-Gómez et al., 2017; Hallsworth and Adams, 2019). The goal for these patients is to prevent the evolution of NAFLD to its advanced form of nonalcoholic steatohepatitis (NASH) which is mainly related to the development of cirrhosis and hepatocellular carcinoma.

Semaglutide, which is a human glucagon-like peptide-1 receptor agonist (GLP-1 RA), seems an attractive therapeutic option for these patients. After binding to its ligand, the GLP-1 receptor activates intracellular signaling pathways that have multiple effects. It is known that semaglutide exerts beneficial effects on parameters of metabolic syndrome, which is directly associated with NAFLD (Eslam et al., 2020; Nauck and Quast, 2021; Gofton et al., 2023; Machado and Cortez-Pinto, 2023). It lowers glucose levels by stimulating glucose-dependent insulin secretion and by reducing fasting and postprandial glucagon (Pyke et al., 2014; Knudsen and Lau, 2019). It also reduces energy intake by affecting appetite (Blundell et al., 2017; Friedrichsen et al., 2021; Gibbons et al., 2021) and inhibits gastric emptying causing weight loss (Brierley et al., 2021; Drucker, 2022). The prevalence of NAFLD in the general population is 30% (Younossi et al., 2023) but in obese patients it is estimated that it ranges from 60% to 95% (Godoy-Matos et al., 2020). Several studies have shown that weight loss is associated with NAFLD resolution (Vilar-Gomez et al., 2015; European Association for the Study of the Liver EASLEuropean Association for the Study of Diabetes EASDEuropean Association for the Study of Obesity EASO, 2016) and semaglutide has already been approved for the management of obesity (Wilding et al., 2021). Moreover, semaglutide reduces cardiovascular risk by decreasing blood pressure, postprandial lipid levels and inflammation (Marso et al., 2016; Rakipovski et al., 2018; Weghuber et al., 2022; Kosiborod et al., 2023).

Nevertheless, apart from the weight loss related benefit in the liver, it has been shown that semaglutide also has antioxidative effects, while it reduces mitochondrial damage, which is considered to play a central role in the pathogenesis and progression of NAFLD (Paradies et al., 2014; Niu et al., 2022). Additionally, data from animal models support the anti-inflammatory effects of semaglutide by inhibition of upregulation of pro-inflammatory factors such as tumor necrosis factor-A and interleukin −6 and by down-regulating the expression of inflammatory factors such as arachidonic acid (Niu et al., 2022). Furthermore, semaglutide reduces lipogenesis and lipid deposition and increases beta-oxidation. All these mechanisms of action are supposed to act beneficially in the improvement of liver histology and NAFLD resolution (Pontes-da-Silva et al., 2022).

So far there are a few literature data regarding the role of semaglutide in NAFLD, mainly from studies concerning type II diabetes mellitus (T2DM) or obesity. In this review, we will focus on the new data concerning the use of semaglutide in NAFLD/NASH patients, i.e., in combination with other medication against NAFLD, as well as its weekly and per os administration.

## Semaglutide in combination therapy

The safety and tolerability of subcutaneous semaglutide alone or in combination with cilofexor (a nonsteroidal farnesoid X receptor agonist) and/or firsocostat (an acetyl-CoA carboxylase inhibitor) in NASH patients with mild-to moderate fibrosis (F2-F3) on biopsy or fat fraction ≥10% on magnetic resonance imaging proton density fat fraction (MRI-PDFF) and liver stiffness ≥7 kPa on transient elastography were evaluated in a phase II open-label, randomized trial by Alkhouri et al. (2022). Patients were treated with semaglutide alone 0.24 mg–2.4 mg once weekly (dose escalated over 16 weeks) or combined with cilofexor 30 mg/day or cilofexor 100 mg/day or firsocostat 20 mg/day or cilofexor 30 mg and firsocostat 20 mg for 24 weeks. Notably, although weight loss was observed in all groups compared to baseline, only the combination of semaglutide plus cilofecor 30 mg achieved greater weight loss compared to semaglutide alone (p<0.05). Liver steatosis (evaluated by MRI-PDFF) was decreased to a greater grade with all combination treatments compared with semaglutide alone, but the improvement was statistically significant only in the semaglutide plus firsocostat arm (−11% vs. −8% in semaglutide alone, *p* = 0.035). Nevertheless, in a sensitivity analysis, excluding patients with imaging data at least 1 month after the last dose, the difference in steatosis between semaglutide alone and semaglutide plus cilofexor plus firsocostat was also significant (−8.6% vs. −12.6%, *p* = 0.008). Interestingly, a greater proportion of patients under combination treatment, compared to semaglutide alone, achieved a relative reduction in MRI-PDFF of ≥50% compared to baseline (58.8%–76.2% vs 38.9%, respectively, always *p* > 0.05). Treatment with semaglutide plus firsocostat and semaglutide plus cilofexor 30 mg significantly reduced liver steatosis assessed by the controlled attenuation parameter (CAP), compared to semaglutide monotherapy (*p* = 0.003 and 0.038, respectively). However, it should be mentioned that all these results regarding liver steatosis were not adjusted with weight loss. Finally, no differences in liver stiffness measured by magnetic resonance elastography (MRE) were observed between groups at the end of study Table 1.

**TABLE 1:** Studies regarding semaglutide in combination treatment and orally in NAFLD/NASH.

REFERENCE/TYPE of study (ref)	Medication	Number of patients/treatment duration (wks)	Effects on liver fibrosis	Effects on liver inflammation and/or steatosis	Effects on liver enzymes	Changes in anthropometric parameters	Changes in laboratory values	Changes in other metabolic parameters	Effects on scores/indexes related to NAFLD	Outlined effects
Alkhouri et al. (2022)/Randomized phase 2 trial	Sema 0.24 mg–2.4 mg once weekly subcutaneously (dose escalated over 16 weeks) alone vs. sema with firsocostat or cilofexor 30 mg or cilofexor 100 mg or firsocostat + cilofexor	108/24	No significant differences in ↓ of liver stiffness (ELF score or transient elastography) from baseline between groups. No differences in liver stiffness (MRE) between groups at the end of study	Sema + firsocostat and sema + cilofexor 30 mg ↓ liver steatosis (CAP), compared to sema monotherapy (*p* = 0.003 and *p* = 0.038 respectively). Greater ↓in liver steatosis (MRI -PDFF) with combo therapies compared with sema alone -significant only for sema + firsocostat arm (*p* = 0.0353)	Greater ↓of ALT with combo therapy compared to sema alone (*p* < 0.05)	↓ of body weight in sema + cilofexor 30 mg group. Similar relative ↓ of body weight from baseline to wk 24 across groups	↓of fasting glu with sema + cilofexor 100 mg. Similar changes from baseline in HbA1c across group. Fircosostat containing regimens ↑ TGs and VLDL but ↓ HDL chol (*p* < 0.05 vs. sema monotherapy).↑LDL at wk 24 with sema + cilofexor 100 mg (*p* < 0.05 vs. sema alone), no changes with cilofexor 30 mg	NA	↓FAST score in all combo regimens except for sema + cilofexor 100 mg compared to sema alone. No differences in ↓of Fibrosure and Fibrotest between combo treatment and monotherapy	Combinations were well tolerated. Combination treatments resulted in greater improvement in hepatic steatosis, liver biochemistry and several hepatic and metabolic parameters compared to sema monotherapy
Arai et al. (2022) Single-arm, open-label pilot study	Oral sema -3 mg daily gradually ↑ to 7 mg at 4 weeks and 14 mg at 8 weeks till the end of study	16/24	No significant improvement in liver stiffness	↓CAP values from baseline to 24 weeks (*p* < 0.01)	↓ALT (*p* < 0.01), AST, γ-GT (*p* < 0.001) from baseline to 12 weeks till 24 weeks	↓body weight and BMI from baseline to 12 weeks till 24 weeks (*p* < 0.001)	↓plasma glu (*p* < 0.01) and HbA1c (*p* < 0.001) from baseline to 12 weeks till 24weeks↓ serum TGs at 24 weeks (*p* < 0.05). ↓ferritin (*p* < 0.01), and type IV collagen 7 (*p* < 0.05) from baseline to wk 24	NA	↓ HOMA-IR and fib-4 index from baseline to wk 24 (*p* < 0.01)	Oral sema in pts with NAFLD complicated by T2DM improved impaired liver function, hypertriglyceridemia, insulin resistance, and hepatic steatosis, as well as improving diabetic status and reducing body weight

NASH, nonalcoholic steatohepatitis, sema, semaglutide, wk, week, vs. *versus*; ELF, enhanced liver fibrosis; MRE, magnetic resonance elastography, combo, combination; CAP, controlled attenuation parameter, MRI PDFF, magnetic resonance imaging proton density fat fraction; ALT, alanine aminotransferase, glu, glucose, HbA1c, hemoglobin A1c, LDL, low density lipoprotein; VLDL, very low density lipoprotein; HDL, high density lipoprotein; TGs, triglycerides, chol, cholesterol, NA, not applicable; FAST, fibroscan-AST, pts, patients; AST, aspartate aminotransferase; γGT, gamma glutamyltransferase; BMI, body mass index; HOMA-IR, homeostasis model assessment—insulin resistance, fib, fibrosis.

## Weekly administration of semaglutide

Newsome et al. (2019) conducted a *post hoc* analysis using data from two randomized, double-blind trials regarding the effects of semaglutide on alanine aminotransferase (ALT) and high sensitivity C reactive protein (hs CRP) in patients who were at risk for NAFLD. The authors analyzed data from a 104‐weeks cardiovascular outcomes trial in T2DM patients with hemoglobin A1c (HbA1c) ≥7% (semaglutide 0.5 or 1.0 mg/week) and a 52‐weeks weight management trial in obese patients without T2DM (semaglutide 0.05–0.4 mg/day). Elevated baseline ALT was recorded in 41% (1,325/3,268) in cardiovascular outcomes-trial subjects, but at the end‐of‐treatment a statistically significant reduction in ALT was seen in the 1.0 mg dose of semaglutide (*p* = 0.0024). However, after adjustment for change in body weight, treatment ratios vs. placebo for ALT and hsCRP were not significant, indicating that these improvements were associated with weight loss Table 2.

**TABLE 2:** Studies regarding weekly semaglutide subcutaneously in NAFLD/NASH.

REFERENCE/TYPE of study	Medication	Number of patients/treatment duration	Effects on liver fibrosis	Effects on liver inflammation and/or steatosis	Effects on liver enzymes	Changes in anthropometric parameters	Changes in laboratory values	Changes in other metabolic parameters	Effects on scores/indexes related to NAFLD	Outlined effects
Newsome et al. (2019)/Data from two trials	Sema 0.05, 0.1, 0.2, 0.3 or 0.4 mg daily/sema 0.5 or 1.0 mg once a wk	957 and 3,297/52 weeks and 104 weeks	NA	NA	↓ ALT 6%–21% (*p* < 0.05 for doses ≥0.2 mg/day)/↓ ALT in the 1.0 mg dose (9% vs. placebo p 0.0024) Reductions also with 0.5 mg at wk 30 not sustained to wk 56	NA	↓ hs CRP 25%–43% (*p* < 0.05)/NA	↓ ∼ 50% of metabolic syndrome prevalence	NA	Sema significantly reduced ALT and hs CRP in clinical trials in subjects with T2DM and/or obesity
Davies et al. (2021)/Double-blind, double-dummy phase 3 superiority study	0nce a week sc administered sema 2.4 mg vs. sema 1.0 mg vs. placebo	1,210 (1,164 completed the trial)/68 weeks	NA	NA	Ratio to baseline at wk 68 (sema 2.4 mg vs. sema 1.0 mg vs. placebo): ALT: 0.74 vs. 0.76 vs. 0.85 AST: 0.88 vs. 0.89 vs. 0.93 γGT: 0.71 vs. 0.75 vs. 0.86	↓ body weight (sema 2.4 mg vs. placebo) wk 68 ETD −6·21 *p* < 0.01. Estimated change in mean bodyweight from baseline to week 68: 9.6% (sema 2.4 mg) vs. −3.4% (placebo)	Sema 2.4 mg vs. placebo wk 68: ↓ HbA1_C_ ETD −1.2 *p* < 0.0001, ↓ total chol ETR: 0.99, ↓ TGs ETR: 0.86, ↓CRP ETR: 0.61	↓systolic BP (sema 2.4 mg vs. placebo) ETD −3·4 *p* = 0.0016	NA	Liver enzymes (ALT, AST, γGT) were decreased in the sema 2.4 mg group and in the sema 1.0 mg group; In overweight or obese with T2DM, sema 2.4 mg achieved a clinically meaningful decrease in bodyweight compared with placebo
Okamoto et al. (2021) Retrospective, single-center, cohort study	Sema 0.25 mg once weekly - increased to 0.5 mg once weekly after 4 weeks or to 1.0 mg once weekly if the efficacy of 0.5 mg once weekly for ≥4 weeks was insufficient	50 (43 switched to sema, 7 naïve to sema)/6 months	NA	NA	Reductions in AST, ALT and γGT (*p* < 0.01)	Switched to sema group: Decreases of body weight at 3 and 6 months (*p* < 0.01). Naïve to sema group: Significant decreases of body weight at 6 months (*p* = 0.02)	Switched to sema group: Decreases of HbA1_C_ at 3 and 6 months (*p* < 0.01); at 6 months total chol and TG levels significantly decreased (*p* < 0.05). Naïve to sema group: At 6 months significant decreases of HbA1C (*p* = 0.04), total chol (*p* = 0.01) and LDL chol (*p* < 0.01)	NA	NA	Sema appears to be more potent in treating type 2 diabetes than existing GLP-1 RAs. Liver related parameters were significantly improved after 6 months of treatment with sema
Volpe et al. (2022)/prospective, single-arm, real-life study	Sema 0.25 mg up to 1 mg weekly	48/52 weeks	NA	Responders to therapy (70%): at least one-class reduction in liver steatosis in the 4-point semiquantitative US staging at the end of study (*p* < 0.001)	↓AST, ALT, γ-GT during the study period compared to baseline (*p* < 0.01)	↓Body weight, body mass index (BMI) and waist circumference after 3, 6 and 12 months of treatment compared to baseline (*p* < 0.01)	After 3 months of therapy, ↓glu, HbA1c, total chol and LDL (*p* < 0.05) (vs. baseline). ↓fasting serum TGs (*p* < 0.05) and insulin levels (*p* < 0.01) after 6 months of therapy till 12 months (vs. baseline). ↑ HDL chol up to 1 year (vs. baseline (*p* < 0.05)	↓US-VAT after 3 months till the end of study (*p* < 0.01) ↓FMI and BIA-VAT after 3,6,12 months (vs. baseline) (all *p* < 0.01). ↓FFMI and SMI from baseline to the end of study (*p* < 0.01). No differences in HG and MQI ↑in SMM/BIA-VAT ratio after 1 year of therapy (vs. baseline) (*p* < 0.01)	↓APRI, HIS. FLI, US-LSS after 3 months till the end of study (*p* < 0.01) ↓(HOMA-IR) index after 6 months of therapy till 12 months compared to baseline (*p* < 0.01)	Besides glucose control and body composition improvements, sema was effective in ameliorating the clinical appearance and severity of NAFLD in T2DM pts
Nomoto et al. (2023) Subanalysis of a multicenter prospective, randomized study	Switch from liraglutide or dulaglutide to weekly sema (SWITCH group) vs. continue liraglutide or dulaglutide (Continue group)	58/24 weeks	ΝΑ	NA	SWITCH group: ↓ALT (*p* = 0.018) and γ-GT (*p* < 0.01) at the end of study. Continue group: No significant changes in these parameters	SWITCH group: Significant ↓in BMI at the end of study compared to baseline (*p* < 0.001). Continue group: No significant difference	SWITCH group: Significant ↓in HbA1c (*p* < 0.05) at the end of study compared to baseline. Continue group: No significant difference	SWITCH group: Significant ↓in total chol (*p* < 0.01) and diastolic BP (*p* < 0.05) at the end of study compared to baseline. Continue group: No significant difference	Improvement of FLI, HIS, ZJU only in the SWITCH group (*p* = 0.002 for FLI and *p* < 0.001 for the rest indexes). Νο improvement in Fib-4 score in the SWITCH group	Switching from conventional GLP-1RAs to once-weekly sema might be beneficial for pts with NAFLD complicated with T2DM
Schattenberg et al. (2023) Analysis of data from two randomized placebo controlled trials	Once weekly sc sema (2.4 mg in STEP 1/1.0 mg and 2.4 mg in STEP 2)	STEP 1:1307 STEP 2:643/68 weeks	ΝΑ	Significantly lower odds of having each NASH component or more severe NAFLD stage at the end of trial for patients who received sema vs. placebo	ΝΑ	ΝΑ	ΝΑ	ΝΑ	NA	Sema had a favourable effect on NASH components in the current analysis in overweight/obese with or without T2DM patients, as measured by SomaSignal models
Loomba et al. (2023) Randomized, placebo-controlled phase 2 trial	Sema 2.4 mg sc once weekly vs. placebo	47 (sema)/24 (placebo) 48 weeks All with cirrhosis	No difference in improvement of liver fibrosis of ≥1 stage without worsening of NASH in liver biopsy after 48 weeks between the two groups. No differences in change of liver stiffness (MRE) between groups	No significant difference for components of NASH or the proportion of pts who achieved NASH resolution (sema vs. placebo). At wk 48, improvement in liver steatosis (MRI-PDFF) (*p* = 0.042) and greater significant reduction in liver fat volume in the sema group (vs. placebo group)	At wk 48, ↓in ALT, AST, γGT from baseline was significantly greater in the sema group vs. the placebo group (p 0.009, 0.046 and 0.037, respectively)	Greater ↓ of body weight from baseline under sema (*p* < 0.0001). Greater proportion of pts achieved a ≥5% (*p* = 0.0047) and ≥10% (*p* = 0.016) weight ↓of body weight at wk 48 with sema vs. placebo. BMI and waist circumference were also significantly lower with sema vs. placebo at wk 48	At wk 48:greater ↓ of HbA1c and glu from baseline in pts with T2DM in the sema group vs. placebo group (*p* < 0.0001 and *p* = 0.001 respectively). Greater ↓ TGs and VLDL chol from baseline with sema vs. placebo (*p* = 0.0013 and *p* = 0.012, respectively)	↓ of BP from baseline under sema at 24 weeks but not different to placebo at 48 weeks	No difference in the improvement of NAS, ELF and SAF score between groups	In pts with NASH and compensated cirrhosis, sema did not significantly improve fibrosis or achievement of NASH resolution vs. placebo. No new safety concerns were raised

NASH, nonalcoholic steatohepatitis; NAFLD, non alcoholic fatty liver disease, sema, semaglutide, wk, week; NA, not applicable; ALT, alanine aminotransferase, vs. *versus*, hs CRP, high sensitivity C- reactive protein, T2DM, type 2 diabetes mellituς, sc, subcutaneous; AST, aspartate aminotransferase; γGT, gamma glutamyltransferase, HbA1c, hemoglobin A1c, OR, odds ratio; ETD, estimated treatment difference, chol, cholesterol; ETR, estimated treatment; BP, blood pressure; LDL, low density lipoprotein; GLP-1, RAs, glucagon like peptide-1 receptor agonists; BMI, body mass index, glu, glucose; HDL, high density lipoprotein; TGs, triglycerides; eGFR, estimated glomelural filtration rate; US-VAT, ultrasound visceral adipose tissue; SAT, subcutaneous adipose tissue; APRI, aspartate aminotransferase to platelet ratio index; HIS, or HSI, hepatic steatosis index; FLI, fatty liver index; ZJU, zhejiang university; LSS, liver steatosis score; FMI, fat mass index; BIA, bioelectrical impedance analysis; FFMI, free fat mass index; SMI, skeletal mass index; HG, hand-grip; MQI, muscle quality index; SMM, skeletal muscle mass; HOMA-IR, homeostasis model assessment—insulin resistance,pts,patients, fib, fibrosis, MRE, magnetic resonance elastography, MRI PDFF, magnetic resonance imaging proton density fat fraction; VLDL, very low density lipoprotein; NAS, NAFLD, activity score; ELF, enhanced liver fibrosis; SAF, steatosis-activity and fibrosis.

Another double-blind, phase III study (Davies et al., 2021) assessed the efficacy and safety of once a week subcutaneously administered semaglutide for weight management in overweight or obese T2DM adults. In total, 1,210 patients were allocated (1:1:1) to receive semaglutide (2.4 mg or 1.0 mg per week) or placebo for 68 weeks. Liver enzymes-ALT, aspartate aminotransferase (AST), gamma glutamyltransferase (γGT)- were evaluated at baseline and week 68. Although no statistical analysis was provided, the authors noted that these liver biochemical parameters -usually increased in the context of NAFLD—reduced from baseline in the semaglutide 2.4 mg/week group as well as in the semaglutide 1.0 mg/week group to a greater extent, compared to placebo group. Thus, the mean ratios to baseline at week 68 for semaglutide 2.4 and 1.0 mg/week vs. placebo were lower for ALT (0.74 and 0.76 vs. 0.85), AST (0.88 and 0.89 vs. 0.93) and γGT (0.71 and 0.75 vs. 0.86). Interestingly, estimated change in mean body weight from baseline to week 68 was –7% with semaglutide 1 mg/week and –9.6% with semaglutide 2.4 mg/week vs. –3.4% with placebo. However, these changes in body weight were not considered for adjustment in liver biochemistry improvement.

A retrospective cohort study (Okamoto et al., 2021) evaluated patients with T2DM who were switched to or initiated on weekly semaglutide because of obesity or poor diabetes mellitus control while treated with other anti-diabetic medications. Forty-three patients were switched to semaglutide (group A) and seven were naïve to semaglutide (group B). Aminotransferases (AST and ALT) and γGT were reduced 6 months after treatment with semaglutide (significant changes in the first group: *p* = 0.01 for AST and ALT, *p* < 0.01 for γGT), compared to the baseline. Interestingly, total cholesterol, triglycerides and uric acid also decreased (statistically significant changes for total cholesterol in both groups, while for triglycerides and uric acid only in the first group). Significant reductions were also noted in ΗbA1c (*p* < 0.01 and *p* = 0.04 for the two groups, respectively), as well as in body weight (p<0.01 in the first group and p = 0.02 in the second one), compared to the baseline. Interestingly, there was no significant correlation between HbA1c and changes in body weight, but similar analysis was not performed regarding aminotransferases reduction.

Another study from Volpe et al. (2022) evaluated the effectiveness of weekly subcutaneous semaglutide add on to metformin in patients with T2DM eligible for glucagon like peptide 1 receptor agonists (GLP-1 RAs). Forty-eight patients received gradually increasing doses of semaglutide (starting from 0.25 mg up to 1 mg per week). Body mass index (BMI) and waist circumference were decreased after three, six and 12 months of treatment compared to baseline (p<0.01) (e.g., mean loss of body weight was 7.4% after 3 months, 9.2% after 6 months and 10.3% at 1 year). Regarding liver biochemistry, AST, ALT, and γ-GT decreased significantly during the study period (*p* < 0.01 compared to baseline). The aspartate aminotransferase to platelet ratio index (APRI) was also significantly reduced after 3 months till the end of study (*p* < 0.01 compared to baseline). Seventy percent of patients -who were defined as responders to therapy-achieved at least one-class reduction in liver steatosis in the 4-point semiquantitative ultrasound (US) staging at the end of study (*p* < 0.001). No adjustment to the weight loss was performed. In the remaining 30% of non-responders, no change in the steatosis grading was found. However, no differences were found between responders and non-responders regarding BMI, HOMA-IR and liver enzymes from baseline to the end of the study.

Interestingly, the fat mass index (FMI) and vascular adipose tissue evaluated by bioelectrical impedance analysis (BIA-VAT) decreased significantly at each observation time after three, six and 12 months compared to baseline (all *p* < 0.01) (Volpe et al., 2022). Although reduction in the skeletal mass index (SMI) was observed, the handgrip (HG) and muscle quality index (MQI) -both indicative of muscular functional status-were not significantly different at the end of study, compared to baseline. In addition, changes in the skeletal muscle mass (SMM)/BIA-VAT ratio progressively increased, reaching significantly higher values than at baseline after 1 year of therapy (*p* < 0.01) (Volpe et al., 2022).

A recently published study from Japan (Nomoto et al., 2023) constituted a sub analysis of a multicenter prospective, randomized study, which compared the efficacy of switching from liraglutide or dulaglutide to once weekly semaglutide on glycemic control in adults with T2DM (SWITCH group) compared to continuing current GLP1RAs (Continue group) for 24 weeks. Semaglutide was started at a dose of 0.25 mg and after at least 4 weeks, the dose was increased to 0.5–1.0 mg weekly. A significant reduction was found in ALT (*p* = 0.018) and γ-GT (<0.01), but without considering confounding factors. No changes in the aforementioned parameters were detected in the Continue group. Fatty liver index (FLI), which was the main outcome of the analysis, improved only in the SWITCH group (*p* = 0.002) but not in the Continue group. Switching to semaglutide did not improve liver fibrosis as assessed by FIB-4 index. Patients in whom dulaglutide was changed to semaglutide showed larger improvements in FLI than those who changed from liraglutide. Both switch strategies (from liraglutide to semaglutide and from dulaglutide to semaglutide) resulted in significant reductions in HbA1c and BMI but no significant differences regarding the extent of the reduction was detected between the subgroups.

In another trial that was presented in an abstract form in the last European Association for the Study of the Liver (EASL) Congress (Schattenberg et al., 2023), SomaSignal tests were applied to proteomics data that were derived from two studies-STEP1 and STEP2-that investigated the effect of weekly subcutaneous semaglutide for 68 weeks on weight loss in overweight or obese patients with (STEP2) or without (STEP1) T2DM, compared to placebo. A targeted proteomics signature derived from patients with histologically proven NASH was developed with the NASH Clinical Research Network (SomaSignal tests) to find the relation between the presence and severity of NASH components and changes over time. Proteomics data were available for 1,307/1961 patients from STEP 1 and 643/1,210 patients from STEP 2. At baseline, 43% of patients in STEP 1 had steatosis whereas the prevalence of the other components was 5% or less. In STEP 2, 72% of patients exhibited steatosis, 15% had NASH and 12% had NASH with fibrosis. The odds of having each NASH histological component were significantly lower at the end of trial for patients who received semaglutide compared to placebo (e.g., in STEP 2 study for semaglutide 1.0 mg/week: 0.25 for steatosis and 0.52 for inflammation). Also, semaglutide was associated with significantly lower odds of having a more severe NAFLD stage after treatment compared to placebo, but nor further data were provided.

### Weekly semaglutide in cirrhosis

In a double-blind, placebo-controlled phase II trial by Loomba et al. (2023), 71 patients (75% with diabetes mellitus) from 38 centres in Europe and the United States with biopsy-confirmed cirrhosis caused by NASH and BMI of ≥27 kg/m^2^ were randomly assigned to receive either once-weekly subcutaneous semaglutide 2.4 mg (n = 47) or placebo (n = 24). The primary endpoint was the proportion of patients with an improvement in liver fibrosis of one stage or more without worsening of NASH in liver biopsy after 48 weeks. At the end of study, although in the placebo group a higher proportion of patients met the primary end point compared to the semaglutide group, this difference was not significant (29% vs. 11%, *p* = 0.087). There was also no difference between groups in the proportion of patients who achieved NASH resolution (*p* = 0.29), as well as regarding the components of NASH (steatosis, lobular inflammation, hepatocyte ballooning). However, at week 48, improvement in liver steatosis assessed by MRI-PDFF was greater in the semaglutide group than in the placebo group (*p* = 0.042) and reduction in liver fat volume was also significantly greater in the semaglutide group than in the placebo group. Concerning liver enzymes, at week 48, reductions in ALT, AST and γGT levels from baseline were significantly greater in the semaglutide group vs. the placebo group (*p* = 0.009, 0.046 and 0.037, respectively). A greater reduction in body weight from baseline was found under semaglutide, compared to placebo group (p<0.01). Also, a greater proportion of patients achieved a ≥5% (p = 0.0047) and ≥10% (p = 0.016) loss of body weight at week 48 with semaglutide, compared to placebo. BMI and waist circumference were also significantly lower with semaglutide, compared to placebo at week 48. However, only baseline BMI was considered as confounding factor for the evaluation of beneficial impact of semaglutide on this cohort Table 2.

Finally, eighty nine percent of patients in the semaglutide group vs. 79% in the placebo group reported adverse events-most of them being nausea (45% vs. 17%), diarrhea (19% vs. 8%) and vomiting (17% vs. 0%). Serious adverse events were reported in 3% and 8% respectively, while no changes in hepatic and renal function and no decompensating events or deaths were noted (Loomba et al., 2023).

## Orally semaglutide

The efficacy and safety of oral semaglutide in patients with NAFLD and T2DM was assessed in a single-arm, open-label pilot study (Arai et al., 2022). Sixteen patients were started on oral semaglutide at a dose of 3 mg daily, which was gradually increased to 7 mg at 4 weeks and 14 mg at 8 weeks till the end of the study at 24th week. Body weight, AST, HbA1c, γ-GT, ALT and plasma glucose decreased significantly from baseline to 12 weeks (p<0.001 for the first four parameters, *p* < 0.01 for the last two) and these changes remained until the end of the study. Levels of the HOMA-IR and serum triglyceride were also significantly reduced at 24 weeks (*p* < 0.01 and *p* < 0.05, respectively). Moreover, CAP values decreased from baseline to 24 weeks (*p* < 0.01). Interestingly, changes in body weight were significantly correlated with those in ALT (r = 0.52, *p* < 0.05) and CAP (r = 0.72, *p* < 0.01). Platelet count increased from baseline to 12 weeks (*p* < 0.05), and it was maintained at 24 weeks (*p* < 0.01). Notably, levels of the fibrosis-4 index, ferritin, and type IV collagen 7 were significantly decreased from baseline to week 24 (*p* < 0.01 for the first two parameters and *p* < 0.05 for the last one). However, the liver stiffness measurement was not significantly improved. Most adverse events were mild to moderate gastrointestinal disorders whereas no severe adverse events or deaths were detected Table 1.

### Orally semaglutide in cirrhosis

A multicenter, open-label, parallel-group trial (Bækdal et al., 2018) investigated whether hepatic impairment affects the pharmacokinetics, safety, and tolerability of oral semaglutide. Child-Pugh classification was used to categorize patients into four groups: normal hepatic function (n = 24) and mild (n = 12), moderate (n = 12), or severe (n = 8) hepatic impairment. Mild impairment was referred to Child-Pugh class A (5–6 points), moderate impairment to Child-Pugh class B (7–9 points) and severe impairment to Child-Pugh class C (10–15 points). The patients received once-daily oral semaglutide (5 mg for 5 days and then 10 mg for the next 5 days). Semaglutide plasma concentrations were measured during dosing and for up to 21 days post-last dose. Area under the semaglutide plasma concentration–time curve from 0 to 24 h after the 10th dose (AUC _0–24h,Day10_)-which was the primary end point-as well as maximum semaglutide concentration after the 10th dose (C_max,Day10_) were similar across groups. Also, time to maximum semaglutide concentration (t_max,Day10_) and half-life (t_1/2,Day10_) were not affected by hepatic impairment. Semaglutide was found to be safe in patients with hepatic impairment. Interestingly, 14.3% of patients reported headache, 8.9% dyspepsia, 7.1% vomiting, 7.1% decreased appetite and 5.4% diarrhea. The authors concluded that no dose adjustment of oral semaglutide is warranted in subjects with hepatic impairment.

## Discussion

Semaglutide, which is a GLP1-RA available in subcutaneous and oral forms, is supposed to exert beneficial effects on NAFLD by numerous mechanisms of action rendering it a promising treatment for the disease (Cigrovski Berkovic et al., 2022). It is known that there is a dose dependent response between weight loss and the magnitude of histological improvement in patients with NAFLD (Godoy-Matos et al., 2020), but apart from the weight loss, semaglutide seems to also benefit liver through anti-inflammatory and antioxidative actions (Niu et al., 2022; Lee and Kim, 2023). The direct hepatic lipid metabolism-modulating properties of GLP1-RAs have also been studied in cell culture models of NAFLD (Petrovic et al., 2023).

Several published studies have evaluated the role of semagutide in patients with NAFLD/NASH, in which semaglutide has been given once a day subcutaneously (Newsome et al., 2019; Flint et al., 2021). However, few studies have evaluated its administration in combination with ‘NAFLD specific drugs’ or its weekly subcutaneous and oral administration.

Many trials are ongoing concerning combination treatments in NAFLD/NASH giving the potential of synergistic effects of the used medicines. ‘NAFLD specific drugs’ are part of the studied regimens. Based on the available literature data (Alkhouri et al., 2022), in which semaglutide was administered either alone or combined with cilofexor and/or firsocostat in patients with NASH and mild to moderate fibrosis, semaglutide combined with firsocostat resulted in greater improvement in hepatic steatosis compared to semaglutide monotherapy estimated by MRI-PDFF and CAP, whereas the combination of semaglutide with cilofexor 30 mg improved hepatic steatosis only measured by CAP. Although no differences in liver fibrosis (evaluated by MRE) were found between groups at the end of study, FAST score, which incorporates liver steatosis and stiffness, was reduced with all combination treatments except for semaglutide plus cilofexor 100 mg. It is well-established that the stage of liver fibrosis is the strongest predictor for development of metabolic-associated comorbidities and liver-related mortality in patients with NASH (Ekstedt et al., 2015; Dulai et al., 2017; Leung et al., 2017). However, no powerful data regarding fibrosis improvement from semaglutide alone or in combination therapy emerged from this study (Alkhouri et al., 2022). Nevertheless, this may be attributed to the short duration of follow up, which was only 6 months. It is of interest that similar weight loss was observed across the study groups indicating that the greater improvements in aminotrasferases, liver fat and FAST with combination therapies were not mediated solely by the loss of body weight and support the complementary actions of farnesoid X receptor agonists and acetyl-coenzyme A carboxylase inhibitors with semaglutide. Regarding the safety of semaglutide, the severity of most of the adverse events were grade 1 or 2, similar across the groups, and thus no drug-drug interactions were clinically observed. However, it should be mentioned that although 108 patients were included in total, each group had a small number of patients, and no placebo control group was incorporated in this study. Additionally, patients with cirrhosis were excluded, but it would be interesting to be enrolled in studies of combination treatment, as data are lacking. Further results from ongoing studies on combination treatments (NCT04971785, NCT05016882, NCT04639414) are awaited.

Very few studies in the literature have evaluated the subcutaneous administration of semaglutide once a week in patients with NAFLD (Newsome et al., 2019; Davies et al., 2021; Okamoto et al., 2021; Volpe et al., 2022; Loomba et al., 2023; Nomoto et al., 2023; Schattenberg et al., 2023) (Table 2). All relevant studies (Newsome et al., 2019; Davies et al., 2021; Okamoto et al., 2021; Volpe et al., 2022; Loomba et al., 2023; Nomoto et al., 2023; Schattenberg et al., 2023) enrolled patients with T2DM with or without obesity. Notably, only three of these studies (Davies et al., 2021; Loomba et al., 2023; Schattenberg et al., 2023) included placebo group-the first one (Davies et al., 2021) evaluated a large cohort of patients with NAFLD (n = 1,210) and with a relatively long duration of follow up (68 weeks). However, only two trials (Loomba et al., 2023; Schattenberg et al., 2023) included patients with histologically proven NASH- the first one (Loomba et al., 2023) regarding cirrhotic patients. Based on the current literature data, weekly semaglutide was found to reduce liver enzymes (Newsome et al., 2019; Davies et al., 2021; Okamoto et al., 2021; Volpe et al., 2022; Loomba et al., 2023; Nomoto et al., 2023) and to achieve loss of body weight (Davies et al., 2021; Okamoto et al., 2021; Volpe et al., 2022; Loomba et al., 2023; Nomoto et al., 2023). The latter is very important, since weight loss has been associated with improvement of metabolic profile (Wilding, 2014) and the risk of cardiovascular disease, which is the leading cause of morbidity and mortality in patients with NAFLD (Targher et al., 2016). Interestingly, these beneficial effects of semaglutide once a week were also confirmed in two trials, in which patients were switched from other GLP1-RAs to weekly subcutaneous semaglutide (Okamoto et al., 2021; Nomoto et al., 2023), indicating that semaglutide may be the GLP1-RAs of choice in patients with NAFLD. Regarding the study by Volpe et al (Volpe et al., 2022), where 10% body weight reduction was observed, it is worth mentioning that at least 10% of body weight loss is required to see NASH resolution (Vilar-Gomez et al., 2015) and this may be the driving factor behind the benefits of semaglutide. Importantly, only one study (Newsome et al., 2019) evaluated the impact of BMI reduction on ALT improvement indicating that liver biochemistry changes under weekly semaglutide administration were associated with weight loss. Thus, further studies are needed to elucidate further this association. As may be expected, weekly administration of semaglutide has been associated with improvement of liver steatosis based on US assessment (Volpe et al., 2022). However, it is known that US has several limitations in this setting including its low sensitivity, particularly in obese individuals or when <30% of liver parenchyma has steatosis (Ferraioli and Monteiro, 2019). Regarding the impact of administration of weekly semaglutide on severity of histological lesions in the liver, this has been assessed only in NASH-associated compensated cirrhosis (Loomba et al., 2023), with no significant improvement in liver fibrosis or resolution of NASH. Probably the short duration of the study (48 weeks) and the presence of baseline cirrhosis prevented the observation of any benefit on histological lesions, and thus, more data are needed to clarify better this issue.

Semaglutide is the only GLP-1RA that has been approved in an oral form, that is something very important reinforcing the compliance of the patients compared to the injectable forms. In the only available study (Arai et al., 2022), daily oral semaglutide in patients with NAFLD and T2DM given for 24 weeks improved parameters of metabolic syndrome, as well as liver steatosis evaluated by CAP. Body weight and BMI reduction was seen from baseline to week 12 till the end of study. These data taken together with the results from studies on weekly semaglutide support the belief that apart from liver specific benefits of semaglutide, weight loss may be the main motivating factor that benefits liver biochemistry and steatosis. However, no improvement was detected in liver fibrosis estimated by transient elastography and fibrosis markers. The small duration of treatment probably did not allow for changes in liver fibrosis to occur. Interestingly, no severe adverse events or deaths were reported. Larger and with long duration trials, regarding the effect of oral semaglutide on the histological lesions of patients with NAFLD/NASH are needed.

It is worth mentioning that a groundbreaking double-blind, placebo-controlled phase three trial study was recently published (Harrison et al., 2023), that pointed out the safety and liver specific benefits of resmetirom on NAFLD, which is the first drug expected to be approved for the disease. Notably, many of these patients were also taking GLP1-RAs including semaglutide in combination with resmetirom during the study, but no separate data were provided. Nevertheless, future studies concerning the co-administration of resmetirom with semaglutide in patients with NAFLD are needed to elucidate if the combination offers an additional benefit, compared to resmetirom or semaglutide alone. In conclusion, semaglutide seems to exert favourable effects on parameters of metabolic syndrome and is a safe drug even in advanced stages of hepatic impairment (Bækdal et al., 2018; Jensen et al., 2018). Several studies regarding its role in NAFLD showed an improvement of liver steatosis. However, improvement in liver fibrosis constitutes a more difficult target and data for its role in preventing the complications of hepatic impairment are still lacking from the literature.
